# The “Chinese Expert Consensus on the Clinical Application of the Chinese Modified Triplet Combination with Irinotecan (CPT-11), Oxaliplatin (LOHP), Continuous Infusion 5-Fluorouracil, and Leucovorin for Colorectal Cancer”

**DOI:** 10.1093/gastro/goab033

**Published:** 2021-09-23

**Authors:** Yanhong Deng, Chinese Southwest Oncology Group - the Committee of Colorectal Cancer

**Affiliations:** 1 Department of Medical Oncology, The Sixth Affiliated Hospital of Sun Yat-sen University, Guangzhou, Guangdong, P. R. China; 2 Guangdong Provincial Key Laboratory of Colorectal and Pelvic Floor Diseases, Guangdong Institute of Gastroenterology, The Sixth Affiliated Hospital of Sun Yat-sen University, Guangzhou, Guangdong, P. R. China

**Keywords:** colorectal cancer, FOLFOXIRI, China, expert consensus

## Abstract

Colorectal cancer is the second most common malignant tumor in China. The FOLFOXIRI regimen, which combines 5-fluorouracil/leucovorin, oxaliplatin, and irinotecan, is a high-intensity and highly effective chemotherapy regimen. However, the original regimen is poorly tolerated in Chinese patients. In order to promote the standardization and rational application of FOLFOXIRI regimen by clinicians in China, the “Chinese Expert Consensus on the Clinical Application of the Chinese Modified Triplet Combination with Irinotecan (CPT-11), Oxaliplatin (LOHP), Continuous Infusion 5-Fluorouracil, and Leucovorin for Colorectal Cancer” was formulated by the Committee of Colorectal Cancer in Chinese Southwest Oncology Group. Based on the mechanism underlying the combined three drugs and toxicity profile, the dosage of Chinese modified FOLFOXIRI (cmFOLFOXIRI) regimen and the management of adverse reactions are proposed. This consensus recommended that the FOLFOXIRI regimen be used in neoadjuvant, conversion, and palliative therapy for colorectal cancer under specific conditions. This consensus aimed to drive the application of cmFOLFOXIRI in the field of colorectal cancer in order to bring benefits to colorectal cancer patients.

## Background

The incidence of colorectal cancer is increasing every year and has become the second most common malignant tumor in China, with >380,000 new cases/year [1]. Among them, ∼60% of patients have distant metastasis at first diagnosis or during the course of the disease and require chemotherapy. Although target therapy has made some progress and immunotherapy is undoubtedly the first-line choice in colorectal cancer patients with microsatellite instability-high/mismatch repair deficiency (MSI-H/dMMR), cytotoxic chemotherapy is the primary line of treatment, especially in the colorectal cancer population with microsatellite stability/mismatch repair proficiency (MSS/pMMR) [2]. Currently, only three types of cytotoxic drugs are proven effective in colorectal cancer, including fluorouracils (5-fluorouracil and capecitabine), oxaliplatin, and irinotecan. The rational design of drug combinations is yet a promising strategy to prolong survival and improve patients’ quality of life.

The FOLFOXIRI regimen—a combination of 5-fluorouracil (5-FU)/calcium leucovorin, oxaliplatin, and irinotecan—is a highly effective chemotherapy regimen. A number of phase III randomized–controlled trials of advanced colorectal cancer have proven that, compared to patients receiving the traditional FOLFOX or FOLFIRI regimen, patients receiving the FOLFOXIRI regimen have a better objective response rate (ORR), progression-free survival (PFS), and overall survival (OS). Presently, the FOLFOXIRI regimen has become one of the recommended chemotherapy regimens for the treatment of advanced colorectal cancer according to the National Comprehensive Cancer Network (NCCN) guidelines, the European Society for Medical Oncology guidelines, and the Chinese Society of Clinical Oncology (CSCO) guidelines.

However, due to the high incidence of adverse reactions and poor patient tolerance of the FOLFOXIRI regimen, especially in Chinese and other Asian populations, the clinical application of the FOLFOXIRI regimen is limited. In addition, relevant clinical research data are mostly based on European and American populations; however, there is no uniform standard for drug usage and dosage. Therefore, we need to urgently make a unified understanding of how to continue to advance to determine the method of use, adverse reactions, and adjust doses. In order to guide clinicians in China to use the FOLFOXIRI regimen for the treatment of colorectal cancer, the Chinese Southwest Oncology Group organized relevant experts in this field. Based on this systematic review of domestic and foreign literature, objective analysis of relevant research data, combined with clinical experience and adapting to the characteristics of patients in China, a consensus was achieved.

## Applicable population

### FOLFOXIRI in a palliative treatment setting

#### Experts’ recommendation

The Chinese modified FOLFOXIRI (cmFOLFOXIRI) combined with bevacizumab is recommended as the first-line palliative treatment for patients who meet the following criteria: unresectable locally advanced or metastatic disease, MSS/pMMR, age 18–75 years, Eastern Cooperative Oncology Group (ECOG) performance status 0–1 if age is <70 years, or ECOG performance status 0 if age is 71–75 years.

#### Treatment administration

First-line treatment is cmFOLFOXIRI plus bevacizumab and the efficacy is evaluated every four cycles; patients without disease progression after eight cycles enter maintenance therapy (5-FU/LV or capecitabine + bevacizumab). Subsequently, patients who develop disease progression during maintenance treatment enter reinduction or second-line therapy. If the oxaliplatin-related peripheral neurotoxicity is lower than grade 2, cmFOLFOXIRI plus bevacizumab can be recommended as the second-line treatment.

#### Illustration

Among the patients with colorectal cancer in China, the proportion of metastatic disease is ∼30%–40% and the 5-year survival rate of metastatic colorectal cancer patients is only 12%. Falcone *et al.*^3^ carried out a series of prospective clinical trials exploring the efficacy of FOLFOXIRI in advanced colorectal cancer and published the phase I/II study in 2002, which confirmed the efficacy and safety of FOLFOXIRI in advanced colorectal cancer for the first time. In 2007, a phase III multicenter randomized–controlled trial showed that FOLFOXIRI is the first-line treatment of metastatic colorectal cancer; FOLFOXIRI has a significantly higher response rate (RR) than FOLFIRI plus bevacizumab (RR, 60% vs 34%, *P *<* *0.0001) [4].

After entering the era of targeted therapy, the TRIBE study confirmed that the triplet FOLFOXIRI plus bevacizumab significantly improves the outcomes of patients with metastatic colorectal cancer compared with FOLFIRI plus bevacizumab. The median PFS was 12.1 months in the experiment group vs 9.7 months in the control group (*P *=* *0.003) and the ORR was 65% vs 53% (*P *=* *0.006) [5]. This study laid the foundation for FOLFOXIRI plus bevacizumab as the first-line treatment in the palliative treatment of advanced colorectal cancer. Falcone *et al*. [6] further carried out the TRIBE-2 study, which was designed to verify whether the triplet FOLFOXIRI plus bevacizumab (experimental group) was superior to preplanned sequential chemotherapy in mFOLFOX6 and FOLFIRI (control group). The primary endpoint of the study, median PFS2 (defined as the time from randomization to disease progression, according to RECIST 1.1, on any treatment administered after first disease progression or death from any cause), was 19.2 and 16.4 months in the study and control groups, respectively (*P *<* *0.001). The study also showed that FOLFOXIRI induced a tumor response. During the first-line treatment, the ORR was 62% and 50% (*P *=* *0.002), the R0 resection rate was 17% and 12% (*P *=* *0.047), and the PFS was 12.0 and 9.8 months (*P *<* *0.001) in the study and control groups, respectively. The proportions of the two groups who continued second-line treatment were 88% and 82%, and the reintroduction rate of FOLFOXIRI in the second-line treatment of the experimental group was 69%. After the first progression, the PFS of second-line treatment in the two groups was 6.2 and 5.6 months (*P *=* *0.11), and no significant difference was detected in the incidence of grade 3–4 adverse events between the two groups [6]. The result of the present study further laid the foundation for bevacizumab combined with FOLFOXIRI in the first-line palliative treatment, whereas other studies compared the triplet therapy with doublet; all the relevant studies are summarized in Table 1 [4–8].

**Table 1.** Summary of randomized–controlled clinical trials of FOLFOXIRI compared with FOLFIRI/FOLFOX in the first-line treatment of metastatic colorectal cancer

Author (study name, year)	Trial phase	Study population	Treatment arms	No. of patients	Median age (range), years	PS 0 (%)	ORR (%)	PFS (months)	*P*-value	OS (months)	*P*-value
Souglakos *et al.* [7](HORG, 2006)	III	Age ≥18 years ECOG PS 0–2	FOLFOXIRI	138	66 (25–82)	36%	43%	TTP: 8.4	0.17	21.5	0.337
			FOLFIRI	147	66 (39–84)	38%	34%	TTP: 6.9		19.5	
Falcone *et al.* [4](GONO, 2007)	III	Age 18–75 yearsECOG PS 0–2 (18–70 years) ECOG PS 0 (71–75 years)	FOLFOXIRI	122	62 (27–75)	61%	66%	9.8	0.0006	22.6	0.032
			FOLFIRI	122	64 (21–75)	61%	41%	6.9		16.7	
Cremolini *et al.* [5](TRIBE, 2014)	III	Age of 18–75 years ECOG PS 0–2 (18–70 years) ECOG PS 0 (71–75 years)	FOLFOXIRI + bev	252	61 (29–75)	90%	65%	12.1	0.003	31.0	0.054
			FOLFIRI + bev	256	60 (29–75)	90%	53%	9.7		25.8	
Hurwitz *et al.* [8] (STEAM, 2016)	I	Age of 18–75 years ECOG PS 0–2 (18–70 years) ECOG PS 0 (71–75 years)	FOLFOXIRI + bev	93	58 (23–75)	67%	72%	11.7	<0.01	concurrently 34.0 sequentially 28.3	Pc = 0.20 Ps = 0.49
			FOLFIRI + be	95	58 (34–73)	54%	62%	9.5		30.7	
Cremolini *et al.* [6] (TRIBE2, 2020)	III	Age 18–75 years ECOG PS 0–2 (18–70 years) ECOG PS 0 (71–75 years)	FOLFOXIRI + bev	339	60 (53–67)	86%	62%	PFS1: 12.0 PFS2: 19.2	P1 = 0.0002 P2 = 0.0005	27.4	0.032
			FOLFOX + bev	340	61 (52–67)	85%	50%	PFS1: 9.8 PFS2: 16.4		22.5	

FOLFOXIRI, 5-Fluorouracil + Oxaliplatin + Irinotecan; FOLFIRI, 5-Fluorouracil + Irinotecan; FOLFOX, 5-Fluorouracil + Oxaliplatin; bev, bevacizumab; ORR, objective response rate; PFS, progression-free survival; OS, overall survival; ECOG PS, Eastern Cooperative Oncology Group performance status; TTP, time to progress.

Presently, FOLFOXIRI combined with bevacizumab has been recommended by NCCN and CSCO guidelines as the standard first-line treatment for patients with advanced colorectal cancer. Therefore, FOLFOXIRI plus bevacizumab was recommended for patients with metastatic colorectal cancer as the first-line palliative treatment, followed by sequential maintenance and the reintroduction of FOLFOXIRI plus bevacizumab after disease progression. For metastatic left-sided colorectal cancer patients with wild-type *RAS/BRAF*, NCCN recommends anti-epidermal growth factor receptor (EGFR) monoclonal antibody combined with FOLFOX/FOLFIRI chemotherapy as the first-line treatment. However, clinical evidence on whether FOLFOXIRI combined with anti-EGFR can be used as a first-line palliative treatment strategy for this population of patients is insufficient.

#### Treatment recommendations for special populations

Treatment recommendations for special populations: v-raf murine sarcoma viral oncogene homolog B (*BRAF*) mutation was critical in patients with advanced colorectal cancer. Several studies reported that V600E is the most common mutation site in the *BRAF* gene [9]. About 5%–12% of patients with metastatic colorectal cancer have the *BRAF* V600E mutation, which indicates poor prognosis or rapid disease progression [10, 11]. In a subgroup analysis of the TRIBE study, patients with metastatic colorectal cancer with the *BRAF* mutation were treated with FOLFOXIRI + bevacizumab (*n *=* *16) and FOLFIRI + bevacizumab (*n *=* *12). The median PFS and OS of the two groups were 7.5 vs 5.5 months and 19.0 vs 10.7 months, respectively [12]. Therefore, for colorectal cancer patients with the *BRAF* V600E mutation, FOLFOXIRI combined with bevacizumab is more effective than other chemotherapy regimens. Concurrently, NCCN guidelines recommend FOLFOXIRI combined with bevacizumab as the first-line treatment regimen of colorectal cancer patients with the *BRAF* V600E mutation and ECOG PS score 0–1.

### FOLFOXIRI in a conversion treatment setting

#### Experts’ recommendation

cmFOLFOXIRI combined with bevacizumab is recommended as the conversion chemotherapy regimen for patients who meet the following criteria: metastatic colorectal cancer that is initially unresectable but has only liver and/or lung metastasis and/or locally treatable lymph node metastasis, MSS/pMMR, age 18–75 years, and ECOG performance status ≤1 if age <70 years or ECOG performance status 0 if age is 71–75 years. Moreover, cmFOLFOXIRI in combination with anti-EGFR monoclonal antibody (cetuximab) is recommended as the first-choice conversion therapy for left-sided colorectal cancer patients with wild-type *RAS/BRAF*.

#### Treatment administration

Imaging evaluation is performed every four cycles of cmFOLFOXIRI plus targeted agents. The multidisciplinary team (MDT) discusses whether it is acceptable to receive radical/destructive local treatment, including primary tumors and metastases surgery, thermal ablation, and stereotactic radiotherapy. FOLFOX with or without targeted therapy can be recommended to patients who need to undergo radical surgery after treatment and the total duration of the perioperative chemotherapy is 6 months (for those using bevacizumab, the drug is stopped for 6 weeks before the operation and 6–8 weeks after the operation). For patients who have received >12 cycles of conversion chemotherapy and still could not receive radical surgery or other local treatment, as assessed by MDT, maintenance therapy (5-FU/leucovorin or capecitabine with or without targeted therapy) is recommended.

#### Illustration

Liver metastasis occurs in ∼50% of patients with colorectal cancer during the course of the disease, which is one of the leading causes of death [13]. Currently, the NCCN guidelines classify colorectal cancer liver metastases as initially resectable and unresectable, which need to be evaluated and classified based on MDT discussion. The potentially resectable liver metastasis refers to the metastatic liver lesion closely related to the critical structure of the liver. In this case, the satisfactory surgical margin could not be marked or sufficient liver volume could not be retained after the resection, which might be transformed to resectable after chemotherapy. The FOLFOXIRI regimen increased the tumor resection rate for initial unresectable metastatic colorectal cancer compared with the FOLFIRI/FOLFOX regimen (Table 2) [4–8, 14, 15]. In METHEP, 125 patients with initial unresectable or potentially resectable colorectal cancer liver metastases were included [14]. The results showed that compared with standard doublet chemotherapy, FOLFIRINOX showed a high effective rate, with a conversion rate of ≤67% [14]. The OLIVIA multinational, randomized phase II trial included previously untreated patients with initially unresectable liver metastases from colorectal cancer. The results showed that the overall resection rate (R0/R1/R2) of bevacizumab plus FOLFOXIRI was significantly higher than that of bevacizumab plus mFOLFOX6 (61% and 49%, respectively). The R0 resection rates were 49% and 23%, the ORR was 81% and 62%, and the median PFS was 18.6 and 11.5 months, respectively. Although the toxicity of the experimental group is higher than that of the control group, it can be controlled [15].

**Table 2.** Resection rate after FOLFOXIRI vs FOLFIRI/FOLFOX in the first-line treatment of metastatic colorectal cancer in randomized–controlled clinical trials

Author (study name, year)	Trial phase	Study population	Treatment arms	No. of patients	Median age (range), years	PS 0 (%)	CRC patients with LM only (%)	ORR (%)	Resection (%)	R0 resection (%)
Souglakos *et al.* [7] (HORG, 2006)	III	Age ≥18 years ECOG PS 0–2	FOLFOXIRI	138	66 (25–82)	36%	NR	43%	10%	9%
			FOLFIRI	147	66 (39–84)	38%	NR	34%	4%	3%
Falcone *et al.* [4] (GONO, 2007)	III	Age of 18–75 years ECOG PS 0–2 (18–70 years) ECOG PS 0 (71–75 years)	FOLFOXIRI	122	62 (27–75)	61%	34%	66%	NR	All: 15% LM only: 36%
			FOLFIRI	122	64 (21–75)	61%	32%	41%	NR	All: 6% LM only: 12%
Ychou *et al.* [14] (METHEP, 2013)	II	Initially unresectable or not optimally resectable liver metastases	FOLFIRINOX	30	64 (43–74)	77%	100%	73%	67%	30%
			FOLFOX/FOLFIRI	30	63 (43–73)	72%	100%	60%	40%	23%
Gruenberger *et al.* [15] (OLIVIA, 2015)	II	Age ≥18 years ECOG PS 0–1 Initially unresectable liver metastases	FOLFOXIRI + bev	41	63 (32–77)	56%	100%	81%	61%	49%
			FOLFIRI + bev	39	57 (28–80)	80%	100%	62%	49%	23%
Cremolini *et al.* [5] (TRIBE, 2014)	III	Age 18–75 years ECOG PS 0–2 (18–70 years) ECOG PS 0 (71–75 years)	FOLFOXIRI + bev	252	61 (29–75)	90%	23%	65%	NR	15%
			FOLFIRI + bev	256	60 (29–75)	90%	18%	53%	NR	12%
Hurwitz *et al.* [8] (STEAM, 2019)	II	Age of 18–75 years ECOG PS 0–2 (18–70 years) ECOG PS 0 (71–75 years)	FOLFOXIRI + bev	93	58 (23–75)	67%	30%	72%	17%	16%
			FOLFIRI + bev	95	58 (34–73)	54%	28%	62%	8%	6%
Cremolini *et al.* [6] (TRIBE2, 2020)	III	Age of 18–75 years ECOG PS 0–2 (18–70 years) ECOG PS 0 (71–75 years)	FOLFOXIRI + bev	339	60 (53–67)	86%	31%	62%	NR	17%
			FOLFOX + bev	340	61 (52–67)	85%	28%	50%	NR	12%

FOLFOXIRI, 5-Fluorouracil + Oxaliplatin + Irinotecan; FOLFIRI, 5-Fluorouracil + Irinotecan; FOLFOX, 5-Fluorouracil + Oxaliplatin; bev, bevacizumab; ORR, objective response rate; ECOG PS, Eastern Cooperative Oncology Group performance status; CRC, colorectal cancer; LM, liver metastasis; NR, not reported.

In POCHER, the study showed that cetuximab combined with FOLFOXIRI administered as conversion chemotherapy can achieve 60% of the R0 resection rate in patients with initially unresectable liver metastases from wild-type RAS/BRAF colorectal cancer [16]. The FOCULM is a two-group, multicenter, phase II trial, which evaluated the addition of cetuximab to cmFOLFOXIRI as conversion therapy. A total of 101 wild-type BRAF/RAS Chinese patients with initially unresectable colorectal liver-only metastasis and ECOG physical status scores of 0 to 1 were enrolled. The cetuximab plus cmFOLFOXIRI group achieved a significantly higher rate of no evidence of disease (NED) (70.1% vs 41.2%, *P *=* *0.005) and an increased ORR (95.5% vs 76.5%, *P *=* *0.010) compared with the FOLFOXIRI group. PFS and OS were both improved in the cetuximab plus cmFOLFOXIRI group, while the incidence of grade 3 and 4 adverse events was similar between the two groups. As for the reported adverse events of liver function, the incidence of grade 3 or above alanine aminotransferase or aspartate aminotransferase elevation was higher in the combination group than in the chemotherapy-alone group (7.5% vs 5.9%, *P *=* *0.005). Among them, 8 patients underwent R0 resection surgery plus thermal ablation and 18 patients underwent thermal ablation therapy for liver metastases, with no significant adverse events reported [17]. A prospective, randomized, multicenter phase II study (VOLFI study) in Germany showed that for wild-type RAS metastatic colorectal cancer with an ECOG physical status score of 0 to 1, the experimental group (FOLFOXIRI combined with panitumumab) had a higher ORR (85.7% vs 54.5%, *P *=* *0.0013) and secondary resection rate than the control group (FOLFOXIRI) [18]. Further analysis indicated that the tumor RR was related to the location of the tumor. The ORR of the left-sided and right-sided tumors in the experimental group was 90.6% and 60.0% (*P *=* *0.0288), respectively, whereas no significant difference was observed in the control group. In patients with left-sided primary tumors, the ORR of the experimental and control groups was 90.6% and 60.0% (*P *=* *0.0039), respectively, whereas in the right-sided primary tumors, the difference in ORR between the two groups was not statistically significant [18].

In summary, patients with wild-type RAS/BRAF left-sided metastatic colorectal cancer undergoing FOLFOXIRI combined with anti-EGFR therapy could achieve a high ORR and R0 resection rate; this regimen is suitable for patients with an optimal physical status score and an urgent need for tumor regression in conversion therapy. Table 3 summarizes the clinical trials of anti-EGFR monoclonal antibodies (cetuximab or panitumumab) combined with chemotherapy to treat liver metastases from colorectal cancer [16, 17, 19–22].

**Table 3.** Summary of clinical trials of anti-epidermal growth factor receptor monoclonal antibodies (cetuximab or panitumumab) combined with chemotherapy conversion for the treatment of colorectal cancer liver metastases

Author (study name, year)	Treatment arms	No. of patients	ORR (%)	Resection (%)	R0 resection (%)	Median survival time (months)
Study population: initially unresectable liver metastases						
Ji *et al.* [19] (2013)	FOLFOX6 + cet	73	73%	49%	27%	NR
Folprecht *et al.* [20] (CELIM, 2010)	FOLFOX6/FOLFIRI + cet	57	NR	NR	28%	NR
Ye *et al.* [21] (BELIFE, 2013)	mFOLFOX6/FOLFIRI	68	29%	NR	7%	21.0
	mFOLFOX6/FOLFIRI + cet	70	57%	NR	26%	30.9
Hu *et al.* [17] (FOCULM, 2020)	cmFOLFOXIRI	34	77%	29%	21%	33.2
	cmFOLFOXIRI + cet	67	96%	55%	36%	NM
Study population: initially unresectable or not optimally resectable liver metastases						
Folprecht *et al.* [20] (CELIM, 2010)	FOLFOX6 + cet	56	68%	42%	38%	35.8
	FOLFIRI + cet	55	57%	43%	30%	29.0
Carrato *et al.* [22] (PLANET-TTD, 2017)	FOLFOX4 + pan	38	74%	45%	34%^a^	37
	FOLFIRI + pan	39	67%	59%	46%^a^	41
Study population: initially not optimally resectable liver metastases						
Garufi *et al.* [16] (POCHER, 2010)	chrono-IFLO + cet	43	79%	79%	60^a^	37

FOLFOXIRI, 5-Fluorouracil + Oxaliplatin + Irinotecan; FOLFIRI, 5-Fluorouracil + Irinotecan; FOLFOX, 5-Fluorouracil + Oxaliplatin; cet, cetuximab; pan, panitumumab; ORR, objective response rate; NR, not reported; NM, not met.

### FOLFOXIRI in neoadjuvant therapy for advanced colorectal cancer

#### Experts’ recommendation

cmFOLFOXIRI is recommended as a neoadjuvant therapy regimen for patients who meet the following criteria: locally advanced (cT3–cT4 or N2) rectal cancer, age 18–75 years, ECOG performance status ≤1 if age was ≤70 years or ECOG performance status 0 if age was 71–75 years.

#### Treatment administration

Typically, four to six cycles of cmFOLFOXIRI are recommended as the preoperative neoadjuvant, followed by tumor regression evaluation with pelvic-enhanced magnetic resonance imaging (MRI). Chemoradiotherapy is considered according to the MDT discussion and the experience in different sites.

#### Illustration

Neoadjuvant therapy is critical in local tumor shrinkage to ensure tumor R0 resection, reducing micrometastases to reduce the risk of tumor recurrence and increasing the anal-preservation rate to improve patients’ quality of life [23]. A high-intensity FOLFOXIRI regimen in neoadjuvant therapy achieves tumor downstaging and reduces the risk of post-operative recurrence; meanwhile, it reduces the complication rates of surgery and improves the quality of life after surgery. The FORTUNE study is a single-arm phase II clinical trial designed to evaluate the efficacy of preoperative cmFOLFOXIRI plus selective radiotherapy in locally advanced rectal cancer [24]. A total of 106 patients with locally advanced rectal cancer were enrolled in this study and treated with four to six cycles of cmFOLFOXIRI chemotherapy before surgery; selective radiotherapy was considered according to tumor regression. The pathological complete response (pCR) rate reached 20.4% (21/103), the downstage rate (ypT0–2N0M0) reached 42.7% (44/103), and the overall chemotherapy was well tolerated. After cmFOLFOXIRI chemotherapy, 12 patients underwent long-term radiotherapy and chemotherapy, and the pCR rate was 41.7% (5/12). The pCR rate of 92 patients who did not receive long-term radiotherapy, including 2 patients receiving chemotherapy plus short-term radiotherapy, was 17.4% (16/92) [24].

In 2020, the American Society of Clinical Oncology meeting reported the results of the PRODIGE 23 study, which is a multicenter phase III study of mFOLFOXIRI with or without radiotherapy for the treatment of locally advanced rectal cancer. A total of 461 patients with rectal cancer (cT3–4NxM0) were enrolled in the study. Participants in the experimental group were administered with six cycles of mFOLFIRINOX chemotherapy, followed by concurrent long-course chemoradiotherapy and then six cycles of mFOLFOX6 chemotherapy after total mesorectal excision (TME). The participants in the control group received only concurrent long-course chemoradiotherapy and then mFOLFOX6 chemotherapy for 6 months after TME. The preliminary results showed that compared with the control group, the experimental group had a higher pCR rate (27.8% vs 12.1%, *P *<* *0.001), a higher 3-year disease-free survival rate (75.7% vs 68.5%, *P *=* *0.034), and a higher 3-year metastasis-free survival rate (78.8% vs 71.7%, *P *=* *0.017); both regimens were well tolerated [25]. Therefore, FOLFOXIRI neoadjuvant chemotherapy plus selective concurrent chemoradiotherapy is considered an effective and safe treatment strategy for patients with locally advanced rectal cancer. In addition, several clinical trials of FOLFOXIRI for neoadjuvant therapy have been being carried out for rectal cancer (Table 4) [24–27].

**Table 4.** Summary of clinical trials of FOLFOXIRI neoadjuvant therapy for rectal cancer

Author (study name, year)	Trial phase	Study population	Treatment arms	No. of patients	PCR (%)	R0 resection (%)	Local recurrence rate	DFS
Rouanet *et al.* [26] (GRECCAR-4, 2020)	II	Age ≥8 years ECOG PS 0–2 MRI staging T3c-4 or MRF+ >1 cm from the anal verge	FOLFOXIRI 4 cycles	10	10%	100%	0	5-DFS: 80%
			Immediate surgery, if ≥75% tumor volume reduction by MRI	19	58%	100%	0	5-DFS: 89.5%
			Standard radiochemotherapy, if ≥75% tumor volume reduction by MRI	52	13.5%	83%	1.9%	5-DFS: 72.9%
			Standard radiochemotherapy, if <75% tumor volume reduction by MRI	49	20%	88%	7.8%	5-DFS: 72.8%
Zhang *et al.* [24] (FORTUNE, 2019)	II	Age of 18–70 years ECOG PS 0–1 MRI staging T3–4 or N+ <12 cm from the anal verge	cmFOLFOXIRI 4–6 cycles, selective radiation after assessment by MRI	103	20.4%	99.0%	7.8%	2-DFS: 85.6% 3-DFS: 78.9%
Conroy *et al.* [25] (PRODIGE-23, 2020)	III	Age of 18–75 years ECOG PS 0–1 MRI staging T3–4 <15 cm from the anal verge	FOLFOXIRI 6 cycles, then radiochemotherapy, followed by surgery at an interval of 7 weeks	231	27.8%	NR	4.8%	2-DFS: 75.7%
			Capecitabine-based concurrent radiochemotherapy, followed by surgery at an interval of 7 weeks	230	12.1%	NR	7%	3-DFS: 68.5%
Masi *et al.* [27] (TRUST, 2019)	II	Age of 18–75 years ECOG PS 0–2 (18–70 years) ECOG PS 0 (71–75 years) MRI staging: high-risk T3 (MRF+, ≤6 cm from the anal verge, T3c∼3d), T4, or stage III <12 cm from the anal verge	Induction FOLFOXIRI plus bevacizumab for 6 cycles, followed by chemoradiotherapy with capecitabine plus bevacizumab	48	36.4%	97.8%	NR	2-DFS: 80.45%

ECOG PS, Eastern Cooperative Oncology Group performance status; DFS, disease-free survival.

Currently, the 2020 version of the NCCN guidelines recommend that 12–16 weeks of FOLFOXIRI followed by concurrent chemoradiotherapy can be considered neoadjuvant chemotherapy for cT4N + locally advanced rectal cancer [28]. None of the studies has yet reported FOLFOXIRI as the neoadjuvant regimen for colon cancer treatment.

### Usage and dosage of the cmFOLFOXIRI regimen

cmFOLFOXIRI consists of an oxaliplatin 85 mg/m^2^ intravenous infusion over 120 min, irinotecan 150–165 mg/m^2^ over 90 min, folinic acid 400 mg/m^2^ over 120 min, and 5-FU 2,400–2,800 mg/m^2^ over a 46–48 h continuous infusion on day 1 every 2 weeks.

For targeted therapy, bevacizumab 5 mg/kg was administered by intravenous infusion on day 1 every 2 weeks. It should be given over 90 min for the first infusion and then over 30–60 min every 2 weeks. Cetuximab 500 mg/m^2^ was administered by intravenous infusion on day 1 every 2 weeks; this intravenous infusion should be given for >120 min for the first time followed by >60 min.

Since the Asian population is less tolerable than the European population, the clinical application of the standard FOLFOXIRI regimen for colorectal cancer in China, even in the entire Asian region, is limited. The results of a phase I dose-finding study showed that the maximum tolerated dose of FOLFOXIRI in Japanese patients with advanced colorectal cancer is 165 mg/m^2^ for irinotecan, 85 mg/m^2^ for oxaliplatin, and 2,400 mg/m^2^ for 5-FU. Thus, the recommended dose of FOLFOXIRI is 150 mg/m^2^ of irinotecan, 85 mg/m^2^ of oxaliplatin, and 2,400 mg/m^2^ of 5-FU [29]. In nine patients with evaluable lesions, the ORR reached 89% and the overall toxicity is manageable. In China, Deng’s group modified the FOLFOXIRI regimen based on the characteristics of the Chinese population, the mechanism of the combined three drugs, and the data of previous studies. Platinum is a dose-dependent drug for efficacy and the dose should not be adjusted, while 5-FU has a wide range of applications and a low value is recommended. An overlap of toxicity of fluorouracil and irinotecan indicated that the dose of irinotecan was reduced by 20%. Thus, a cmFOLFOXIRI regimen indicated that the dose of irinotecan was reduced from 180 mg/m^2^ in the original regimen to 150–165 mg/m^2^; 5-FU was reduced from 3,200 mg/m^2^ to 2,400–2,800 mg/m^2^. Another retrospective study enrolled 113 patients with locally advanced colorectal cancer and 199 patients with metastatic colorectal cancer who received cmFOLFOXIRI with or without a targeted therapy (bevacizumab or cetuximab) regimen. For patients with metastatic colorectal cancer, the disease-control rate was 93.8%, the ORR was 63.8%, and the partial RR reached 61.6%. Regarding the safety of patients, the incidence rates of grade 3/4 neutropenia, fatigue, and anemia were 22.1%, 11.2%, and 8.9%, respectively, and the incidence of serious adverse events, including febrile neutropenia and intestinal perforation, was 6.4% [30]. The FORTUNE and FOCULM studies further verified that reducing the dose is beneficial to clinical management [17, 24]. The adverse events of cmFOLFOXIRI are significantly lower than those reported in the TRIBE and OLIVIA trials. However, the efficacies of cmFOLFOXIRI and TRIBE or OLIVIA are equivalent. Therefore, cmFOLFOXIRI is suitable for the Chinese population. Currently, the TRIBE-C (ClinicalTrials.gov, NCT04230187) trial is ongoing to evaluate the efficacy and safety of cmFOLFOXIRI combined with bevacizumab for advanced colorectal cancer compared with those of mFOLFOX6 combined with bevacizumab, which aimed to prove the efficacy of dose-modified cmFOLFOXIRI in patients with metastatic colorectal cancer in China.

### Adverse events of cmFOLFOXIRI: prevention and management

cmFOLFOXIRI has a manageable safety profile in colorectal cancer after dose modification. The common grade ≥3 adverse events include neutropenia, diarrhea, and fatigue. The dose intensity of the FOLFOXIRI regimen used in the TRIBE-2 study might be extremely high. In terms of adverse events in the TRIBE-2 study, the most common grade ≥3 adverse events in the FOLFOXIRI group during first-line treatment were diarrhea (17%) and neutropenia (50%) [6]. However, the occurrence rate of grade ≥3 neutropenia in the Chinese colorectal cancer population with cmFOLFOXIRI was 42.5% and the occurrence rate of diarrhea, fatigue, and nausea and vomiting were only 2%, 3.8%, and 8.5%, which were lower than those of the standard dose of FOLFOXIRI. Thus, the completion rate of chemotherapy was improved [24, 30]. In addition, the occurrence rate of common adverse events of cmFOLFOXIRI combined with targeted therapy was not significantly increased compared with that in cmFOLFOXIRI alone. In FOCULM, except for the significantly higher occurrence rate of acne-like rash (55.2% vs 5.9%, *P *<* *0.001), the occurrence rate of other adverse events did not increase in cmFOLFOXIRI combined with cetuximab compared with that in cmFOLFOXIRI alone [17].

The risks of patients were assessed and the adverse events with respect to the clinical application of cmFOLFOXIRI were identified. In addition, the metabolic enzyme UGT1A1 in the liver and intestines could glucuronidate the active molecule SN-38 of irinotecan, thereby reducing the adverse events [31]. Some studies confirmed that UGT1A1*28 and UGT1A1*6 are related to chemotherapy-related diarrhea and neutropenia induced by irinotecan [32, 33]. In 2015, the US FDA considered UGT1A1*28 polymorphism as a predictor of irinotecan-induced toxicity. In the Asian population, the mutation frequency of UGT1A1*28 gene is lower, whereas the mutation frequency of UGT1A1*6 is higher [34]. Therefore, UGT1A1 could be tested before irinotecan chemotherapy and guide dose adjustment and drug selection. Although the adverse events of the cmFOLFOXIRI regimen are manageable after dose modification, they are still higher than those of the FOLFOX/FOLFIRI regimen. During treatment, patients’ adverse events should be closely monitored and the dose modification after grade 3 toxicities (NCI CTCAE 5.0) is summarized in Table 5. Specific attention should be paid to adverse events, such as fatigue, neutropenia, diarrhea, intestinal obstruction, and intestinal perforation (Table 6 ).

**Table 5.** Dose adjustment of common adverse events of the cmFOLFOXIRI regimen

Adverse event	Grade	Irinotecan	Oxaliplatin	5-Fluorouracil
Neutropenia	Grade 4	80%	80%	100%
Febrile neutropenia	Grade 3/4	80%	80%	100%
Thrombocytopenia	Grade 3/4	80%	80%	100%
Alanine aminotransferase	Grade 3/4	80%	80%	100%
Aspartate aminotransferase	Grade 3/4	80%	80%	100%
Serum total bilirubin	Grade 3/4	80%	80%	100%
Diarrhea	Grade 3	80%	100%	80%
Diarrhea	Grade 4	60%	100%	80%
Oral mucositis	Grade 3	80%	100%	80%
Oral mucositis	Grade 4	80%	100%	60%
Myocardial infarction	Grade 3/4	100%	100%	STOP

In the case that the adverse event meets neither the standard of dose reduction nor the standard of administration for the prescribed time of the next cycle, which occurs twice or more repeatedly, following which the decline in the dose. If the dose was reduced twice, the drug should be discontinued. The original dose in this table refers to the dose of the previous chemotherapy, all of which was reduced based on the previous dose.

**Table 6.** Management strategies for adverse events requiring special attention

Adverse event	Grade 3–4 (%) [24, 30]	Recommendations
Neutropenia	22.2%–42.5%	Hematopoietic functions in baseline were evaluated before treatment for every patient Prophylactic use of filgrastim (G-CSF) or sargramostim (GM-CSF) is recommended In addition to bone marrow suppression, splenomegaly and hypersplenism should be focused on, and local treatment of the spleen if necessary Dose adjustment of chemotherapeutic drugs is required for grade 4 neutropenia Occurrence of febrile neutropenia requires dose adjustment and therapeutic antibiotics for infections
Diarrhea	2%–6%	Early-onset diarrhea usually occurs on the day of medication, mainly accompanied by cramping abdominal pain, tearing, sweating, increased saliva, hypotension, dizziness, and blurred vision. In severe cases, atropine 0.25 mg can be injected subcutaneously, along with intravenous rehydration therapy Delayed diarrhea: dose-limiting toxicity, which usually appears after 24 h of medication, with an incidence of 80%–90%, of which 39% are severe. The median time of occurrence was 5 days after medication, lasting for an average of 4 days, and severe cases can be fatal. Loperamide (Imodium) can be administered orally: the first dose is 4 mg and then 2 mg every 2 h. Continue to use the medication for 12 h after the last watery defecation and generally do not exceed 48 h. If diarrhea persists for >48 h, seek medical attention immediately Dose adjustment of chemotherapeutic drugs is required for grade 3–4 diarrhea
Fatigue	3.8%–11.2%	Evaluate the patient’s nutritional status before treatment and intervene Add nutritional support during treatment Strengthen health survey
Intestinal obstruction	2.9%–4.8%	Evaluate the size of the primary tumor and the risk of obstruction before treatment, and use laxatives if necessary to keep the bowel open Pay attention to the use of antiemetic drugs during treatment. Long-acting 5-HT antagonists are not recommended for patients at a high risk of obstruction Deal with difficulties in defecation in time Strengthen dietary guidance If intestinal obstruction occurs during treatment and conservative medical treatment is ineffective, preventive stoma surgery could be considered
Intestinal perforation	2.2%	Evaluate the size of the primary tumor and the risk of perforation before treatment, and keep the bowel open Once perforation occurs during treatment, immediately evaluate the operation and provide the best supportive treatment

## Conclusions

The FOLFOXIRI regimen has gradually transferred from the palliative therapy of advanced colorectal cancer to the neoadjuvant therapy of locally advanced colorectal cancer. The application of this regimen in the field of colorectal cancer continues to advance, benefiting colorectal cancer patients. Simultaneously, cmFOLFOXIRI improved the tolerance of the Chinese population by reducing the dose of irinotecan and 5-FU, ultimately improving the efficacy for the patients. Nonetheless, additional studies are required in the future to make the cmFOLFOXIRI regimen precise and customized to improve the efficacy and avoid over-treatment.

## Members of the consensus expert group

Consultants: Houjie Liang (Southwest Hospital Affiliated to Army Medical University), Tongyu Lin (Sichuan Cancer Hospital), Lin Shen (Peking University Cancer Hospital), Jianping Wang (the Sixth Affiliated Hospital of Sun Yat-sen University), Xishan Wang (Cancer Hospital of Chinese Academy of Medical Sciences), Ruihua Xu (Sun Yat-sen University Cancer Center), Ding Yu (Hubei Cancer Hospital), and Suzhan Zhang (the Second Affiliated Hospital, Zhejiang University School of Medicine)

Chairman: Yanhong Deng

Deputy Chairman: Gong Chen, Jian Li, Meng Qiu, Xianglin Yuan, Tao Zhang

Members: Gong Chen (Sun Yat-sen University Cancer Center), Yanhong Deng (the Sixth Affiliated Hospital of Sun Yat sen University), Peirong Ding (Sun Yat-sen University Cancer Center), Fang Weijia (the First Affiliated Hospital, Zhejiang University School of Medicine), Zengqing Guo (Fujian Cancer Hospital), Xiaohua Hu (the First Affiliated Hospital of Guangxi Medical University), Hong Jiang (Tongji Hospital Affiliated to Tongji University), Yongdong Jin (Sichuan Cancer Hospital), Jian Li (Peking University Cancer Hospital), Yong Li (the Second Affiliated Hospital, Guangzhou University of Traditional Chinese Medicine), Qu Lin (the Third Affiliated Hospital, Sun Yat sen University), Rongbo Lin (Fujian Cancer Hospital), Dong Ma (Guangdong Provincial People's Hospital), Lijun Ma (Tongren Hospital, Shanghai Jiaotong University School of Medicine), Hong Qiu (Tongji Hospital, Tongji Medical College, Huazhong University of Science and Technology), Meng Qiu (West China Hospital of Sichuan University), Kun Wang (Peking University Cancer Hospital), Wei Wang (The First People's Hospital of Foshan City), Wenling Wang (Cancer Hospital of Guizhou Province), Xiaozhong Wang (Shantou Central Hospital), Derong Xie (Sun Yat Sen Memorial Hospital, Sun Yat sen University), Lin Xie (Yunnan Cancer Hospital), Ruilian Xu (Shenzhen People's Hospital), Yan Yang (Gansu Cancer Hospital), Xianli Yin (Hunan Cancer Hospital), Yuan Xianglin (Tongji Hospital, Tongji Medical College, Huazhong University of Science and Technology), Yuan Ying (the Second Affiliated Hospital, Zhejiang University School of Medicine), Guoping Zhang (Yuebei people's Hospital), Hongyu Zhang (the Fifth Affiliated Hospital, Sun Yat-sen University), Huiqing Zhang (Jiangxi Cancer Hospital), Jingdong Zhang (Liaoning Cancer Hospital), Jun Zhang (Ruijin Hospital, Shanghai Jiaotong University School of Medicine), Tao Zhang (Union Hospital, Tongji Medical College, Huazhong University of Science and Technology), Yanqiao Zhang (Harbin Medical University Cancer Hospital), Yunbo Zhao (Beijing hospital), Liangjun Zhu (Jiangsu Cancer Hospital), Hong Zong (the First Affiliated Hospital of Zhengzhou University)

Secretary group: Yue Cai, Yi Cheng, Huabin Hu, Jianwei Zhang

## References

[1] Zheng RS, Sun KX, Zhang SW et al Report of cancer epidemiology in China, 2015 [in Chinese]. Zhonghua Zhong Liu Za Zhi 2019;41:19–28. 30678413 10.3760/cma.j.issn.0253-3766.2019.01.005

[2] André T, Shiu KK, Kim TW et al; KEYNOTE-177 Investigators. Pembrolizumab in microsatellite-instability-high advanced colorectal cancer. N Engl J Med 2020;383:2207–18. 33264544 10.1056/NEJMoa2017699

[3] Falcone A, Masi G, Allegrini G et al Biweekly chemotherapy with oxaliplatin, irinotecan, infusional fluorouracil, and leucovorin: a pilot study in patients with metastatic colorectal cancer. J Clin Oncol 2002;20:4006–14. 12351598 10.1200/JCO.2002.12.075

[4] Falcone A, Ricci S, Brunetti I et al Phase III trial of infusional fluorouracil, leucovorin, oxaliplatin, and irinotecan (FOLFOXIRI) compared with infusional fluorouracil, leucovorin, and irinotecan (FOLFIRI) as first-line treatment for metastatic colorectal cancer: the Gruppo Oncologico Nord Ovest. J Clin Oncol 2007;25:1670–6. 17470860 10.1200/JCO.2006.09.0928

[5] Cremolini C, Masi G, Lonardi S et al Initial therapy with FOLFOXIRI and bevacizumab for metastatic colorectal cancer. N Engl J Med 2014;371:1609–18. 25337750 10.1056/NEJMoa1403108

[6] Cremolini C, Antoniotti C, Rossini D et al; GONO Foundation Investigators. Upfront FOLFOXIRI plus bevacizumab and reintroduction after progression versus mFOLFOX6 plus bevacizumab followed by FOLFIRI plus bevacizumab in the treatment of patients with metastatic colorectal cancer (TRIBE2): a multicentre, open-label, phase 3, randomised, controlled trial. Lancet Oncol 2020;21:497–507. 32164906 10.1016/S1470-2045(19)30862-9

[7] Souglakos J, Androulakis N, Syrigos K et al FOLFOXIRI (folinic acid, 5-fluorouracil, oxaliplatin and irinotecan) vs FOLFIRI (folinic acid, 5-fluorouracil and irinotecan) as first-line treatment in metastatic colorectal cancer (MCC): a multicentre randomised phase III trial from the Hellenic Oncology Research Group (HORG). Br J Cancer 2006;94:798–805. 16508637 10.1038/sj.bjc.6603011 PMC2361370

[8] Hurwitz HI, Tan BR, Reeves JA et al Phase II randomized trial of sequential or concurrent FOLFOXIRI-bevacizumab versus FOLFOX-bevacizumab for metastatic colorectal cancer (STEAM). Oncologist 2019;24:921–32. 30552157 10.1634/theoncologist.2018-0344 PMC6656450

[9] Jolien T, Nagtegaal ID, Punt CJA. BRAF mutation in metastatic colorectal cancer. N Engl J Med 2009;361:98–9. 10.1056/NEJMc0904160 19571295

[10] Yokota T, Ura T, Shibata N et al BRAF mutation is a powerful prognostic factor in advanced and recurrent colorectal cancer. Br J Cancer 2011;104:856–62. 21285991 10.1038/bjc.2011.19 PMC3048210

[11] Richman SD, Seymour MT, Philip C et al KRAS and BRAF mutations in advanced colorectal cancer are associated with poor prognosis but do not preclude benefit from oxaliplatin or irinotecan: results from the MRC FOCUS trial. J Clin Oncol 2009;27:5931–7. 19884549 10.1200/JCO.2009.22.4295

[12] Cremolini C, Loupakis F, Antoniotti C et al FOLFOXIRI plus bevacizumab versus FOLFIRI plus bevacizumab as first-line treatment of patients with metastatic colorectal cancer: updated overall survival and molecular subgroup analyses of the open-label, phase 3 TRIBE study. Lancet Oncol 2015;16:1306–15. 26338525 10.1016/S1470-2045(15)00122-9

[13] Zarour L, Anand S, Billingsley K et al Colorectal cancer liver metastasis: evolving paradigms and future directions. Cell Mol Gastroenterol Hepatol 2017;3:163–73. 28275683 10.1016/j.jcmgh.2017.01.006 PMC5331831

[14] Ychou M, Rivoire M, Thezenas S et al A randomized phase II trial of three intensified chemotherapy regimens in first-line treatment of colorectal cancer patients with initially unresectable or not optimally resectable liver metastases: the METHEP trial. Ann Surg Oncol 2013;20:4289–97. 23955585 10.1245/s10434-013-3217-x

[15] Gruenberger T, Bridgewater J, Chau I et al Bevacizumab plus mFOLFOX-6 or FOLFOXIRI in patients with initially unresectable liver metastases from colorectal cancer: the OLIVIA multinational randomised phase II trial. Ann Oncol 2015;26:702–8. 25538173 10.1093/annonc/mdu580

[16] Garufi C, Torsello A, Tumolo S et al Cetuximab plus chronomodulated irinotecan, 5-fluorouracil, leucovorin and oxaliplatin as neoadjuvant chemotherapy in colorectal liver metastases: POCHER trial. Br J Cancer 2010;103:1542–7. 20959822 10.1038/sj.bjc.6605940 PMC2990583

[17] Hu H, Wang K, Huang M et al Modified FOLFOXIRI with or without cetuximab as conversion therapy in patients with RAS/BRAF wild-type unresectable liver metastases colorectal cancer: the FOCULM multicenter phase II trial. Oncologist 2021;26:e90–8. 33400355 10.1634/theoncologist.2020-0563 PMC7794191

[18] Modest DP, Martens UM, Riera-Knorrenschild J et al FOLFOXIRI plus panitumumab as first-line treatment of RAS wild-type metastatic colorectal cancer: the randomized, open-label, phase II VOLFI study (AIO KRK0109). J Clin Oncol 2019;37:3401–11. 31609637 10.1200/JCO.19.01340

[19] Ji JH, Park SH, Lee J et al Prospective phase II study of neoadjuvant FOLFOX6 plus cetuximab in patients with colorectal cancer and unresectable liver-only metastasis. Cancer Chemother Pharmacol 2013;72:223–30. 23689915 10.1007/s00280-013-2190-1

[20] Folprecht G, Gruenberger T, Bechstein WO et al Tumour response and secondary resectability of colorectal liver metastases following neoadjuvant chemotherapy with cetuximab: the CELIM randomised phase 2 trial. Lancet Oncol 2010;11:38–47. 19942479 10.1016/S1470-2045(09)70330-4

[21] Ye LC, Liu TS, Ren L et al Randomized controlled trial of cetuximab plus chemotherapy for patients with KRAS wild-type unresectable colorectal liver-limited metastases. J Clin Oncol 2013;31:1931–8. 23569301 10.1200/JCO.2012.44.8308

[22] Carrato A, Abad A, Massuti B et al; Spanish Cooperative Group for the Treatment of Digestive Tumours (TTD). First-line panitumumab plus FOLFOX4 or FOLFIRI in colorectal cancer with multiple or unresectable liver metastases: a randomised, phase II trial (PLANET-TTD). Eur J Cancer 2017;81:191–202. 28633089 10.1016/j.ejca.2017.04.024

[23] Rodel C, Liersch T, Becker H et al; German Rectal Cancer Study Group. Preoperative chemoradiotherapy and postoperative chemotherapy with fluorouracil and oxaliplatin versus fluorouracil alone in locally advanced rectal cancer: initial results of the German CAO/ARO/AIO-04 randomised phase 3 trial. Lancet Oncol 2012;13:679–87. 22627104 10.1016/S1470-2045(12)70187-0

[24] Zhang J, Huang M, Cai Y et al Neoadjuvant chemotherapy with mFOLFOXIRI without routine use of radiotherapy for locally advanced rectal cancer. Clin Colorectal Cancer 2019;18:238–44. 31378655 10.1016/j.clcc.2019.07.001

[25] Conroy T, Lamfichekh N, Etienne PL et al Total neoadjuvant therapy with mFOLFIRINOX versus preoperative chemoradiation in patients with locally advanced rectal cancer: final results of PRODIGE 23 phase III trial, a UNICANCER GI trial. J Clin Oncol 2020;38:4007.

[26] Rouanet P, Rullier E, Lelong B et al Tailored treatment strategy for locally advanced rectal carcinoma: five-year results of the French phase II, randomized, multicenter GRECCAR4 trial. J Clin Oncol 2020;38. 28594714 10.1097/DCR.0000000000000849

[27] Masi G, Vivaldi C, Fornaro L et al Total neoadjuvant approach with FOLFOXIRI plus bevacizumab followed by chemoradiotherapy plus bevacizumab in locally advanced rectal cancer: the TRUST trial. Eur J Cancer 2019;110:32–41. 30739838 10.1016/j.ejca.2019.01.006

[28] Benson AB, Venook AP, Al-Hawary MM et al NCCN guidelines insights: rectal cancer, version 6.2020. J Natl Compr Canc Netw 2020;18:806–15. 32634771 10.6004/jnccn.2020.0032

[29] Sunakawa Y, Fujita K, Ichikawa W et al A phase I study of infusional 5-fluorouracil, leucovorin, oxaliplatin and irinotecan in Japanese patients with advanced colorectal cancer who harbor UGT1A11/1,*1/*6 or *1/*28. Oncology 2012;82:242–8. 22508373 10.1159/000337225

[30] Cai Y, Deng R, Hu H et al Analysis on safety and preliminary efficacy of dose-modified regimen of 5-fluorouracil plus oxaliplatin and irinotecan (FOLFOXIRI) in advanced colorectal cancer [in Chinese]. Zhonghua Wei Chang Wai Ke Za Zhi 2018;21:1045–50. 30269326

[31] Hahn KK, Wolff JJ, Kolesar JM. Pharmacogenetics and irinotecan therapy. Am J Health Syst Pharm 2006;63:2211–7. 17090741 10.2146/ajhp060155

[32] Minami H, Sai K, Saeki M et al Irinotecan pharmacokinetics/pharmacodynamics and UGT1A genetic polymorphisms in Japanese: roles of UGT1A16 and 28. Pharmacogenet Genomics 2007;17:497–504. 17558305 10.1097/FPC.0b013e328014341f

[33] Martinez-Balibrea E, Abad A, Martínez-Cardús A et al UGT1A and TYMS genetic variants predict toxicity and response of colorectal cancer patients treated with first-line irinotecan and fluorouracil combination therapy. Br J Cancer 2010;103:581–9. 20628391 10.1038/sj.bjc.6605776 PMC2939780

[34] Desai AA, Innocenti F, Ratain MJ. Pharmacogenomics: road to anticancer therapeutics nirvana? Oncogene 2003;22:6621–8. 14528287 10.1038/sj.onc.1206958

